# Shedding light on bacteria–host interactions with the aid of TnSeq approaches

**DOI:** 10.1128/mbio.00390-24

**Published:** 2024-05-09

**Authors:** Marta Torres, Sarah Paszti, Leo Eberl

**Affiliations:** 1 Department of Plant and Microbial Biology, University of Zurich, Zürich, Switzerland; Instituto Carlos Chagas, Curitiba, Brazil

**Keywords:** host–bacteria interactions, colonization, fitness genes, transposon sequencing

## Abstract

Bacteria are highly adaptable and grow in diverse niches, where they often interact with eukaryotic organisms. These interactions with different hosts span the entire spectrum from symbiosis to pathogenicity and thus determine the lifestyle of the bacterium. Knowledge of the genetic determinants involved in animal and plant host colonization by pathogenic and mutualistic bacteria is not only crucial to discover new drug targets for disease management but also for developing novel biostimulant strategies. In the last decades, significant progress in genome-wide high-throughput technologies such as transposon insertion sequencing has led to the identification of pathways that enable efficient host colonization. However, the extent to which similar genes play a role in this process in different bacteria is yet unclear. This review highlights the commonalities and specificities of bacterial determinants important for bacteria–host interaction.

## COLONIZATION OF EUKARYOTIC HOSTS AND THE NEED TO UNRAVEL THE MECHANISMS INVOLVED

Bacterial colonization of plant and animal hosts is widespread in nature. Different outcomes of host–bacteria interactions have led to distinguishing between contrasting lifestyles or relationships: (i) mutualism, when benefits are obtained by both bacteria and host; (ii) commensalism, when the interaction is beneficial to the bacteria and it is not harming the host; (iii) pathogenicity, when bacteria have the ability to damage the host and cause disease; and (iv) opportunism, when a bacterium that usually does not cause disease becomes pathogenic after perturbation of host defenses (1–3). Mutualism and commensalism are not detrimental for the host, while pathogenicity and opportunism are. Interestingly, some pathogens can switch the lifestyle to a non-detrimental (asymptomatic) state in a certain habitat, which then can serve as infection reservoir (4). Regardless of the outcome of the host–bacteria association, the success of these interactions requires evasion of the host innate immunity and ability to persist in the host for an effective bacterial colonization (5). The terminology of host–bacteria interactions is constantly being debated and thus is often diffuse. Terms such as infection, persistence, commensalism, colonization, symbiosis, and disease are repeatedly used inconsistently and should be re-examined (1, 6). The definition of these concepts, which have been in use in medicine for nearly a century, is normally only focusing on pathogenic bacteria and does not consider mutualistic microorganisms. In this review, we will refer to these terms collectively as “colonization” or “persistence” (i.e., colonization over extended periods of time).

**Fig 1:**
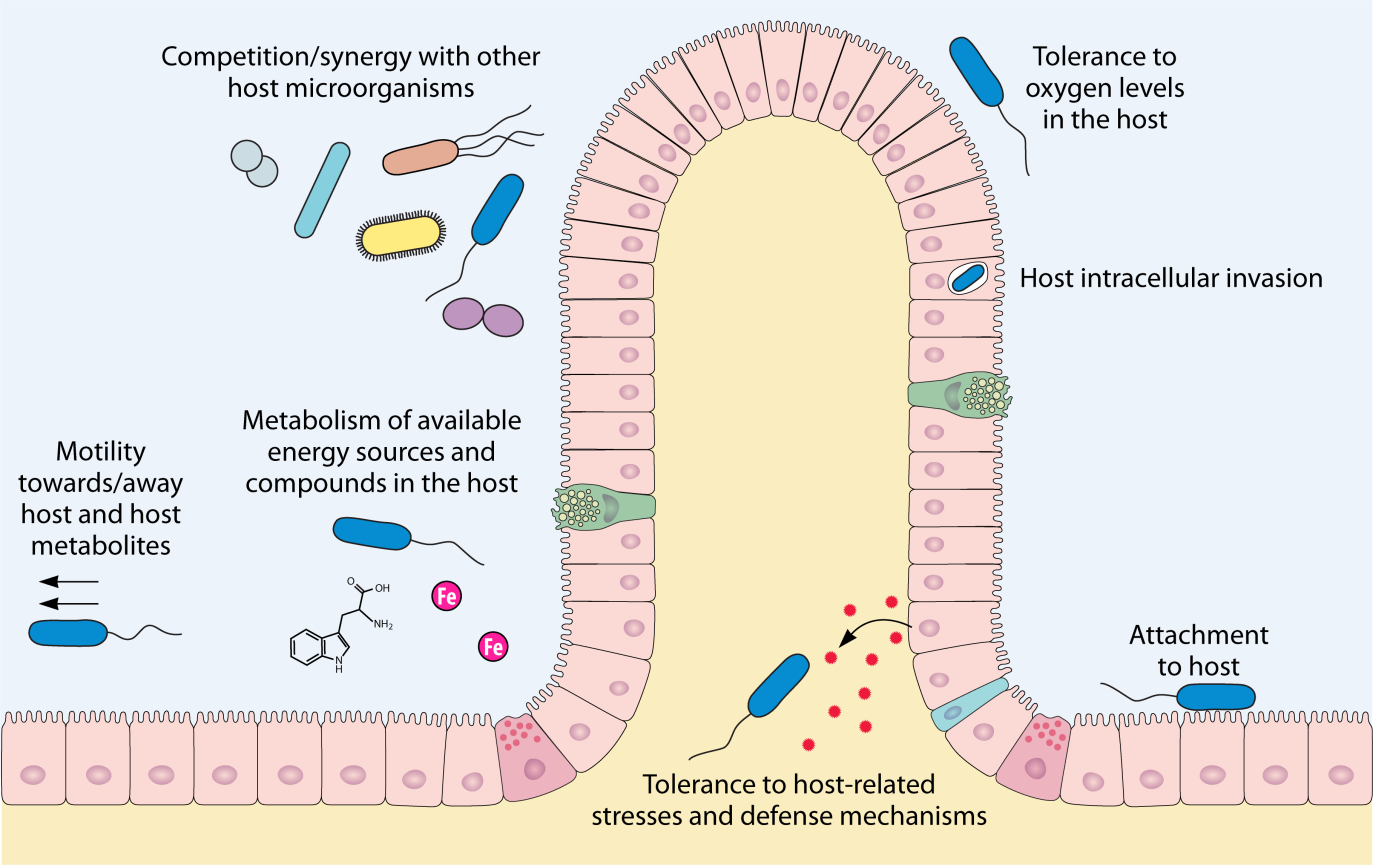
Overview of cell functions involved in host colonization. Examples shown include motility toward/away the host and its metabolites, tolerance to host-related stresses and defense mechanisms, metabolism of available energy sources in the host, attachment, intracellular invasion, and competition/synergy with other host microorganisms, among others.

Some bacteria are adapted to live in specific locations in their hosts, and in many cases, colonization of such sites by other microorganisms is excluded. Other bacteria can colonize a wide range of hosts and host compartments. In both cases, bacterial cells have evolved a wide range of strategies to colonize their hosts. Establishment of bacteria within a host requires various functions such as attachment, biofilm formation, tissue invasion, stress tolerance, motility, nutrient acquisition, metabolic versatility, and competition with other microorganisms, among others (Fig. 1) (3, 7–13). Along with these diverse microbial factors influencing colonization, host immunity and its escape play crucial roles in determining which microbes can colonize and persist (5).

Across different types of interaction (i.e., mutualism and pathogenicity) and types of hosts (i.e., plants and animals), there are aspects of a host environment that are shared. Several studies have shown that, unsurprisingly, closely related bacteria that colonize hosts with diverse outcomes or lifestyles share a number of host association factors (3, 14). There is also evidence which suggests an overlap between colonization determinants of plant and animal hosts (15–17). Both types of hosts belong to the domain Eukarya, even though they each evolved independently into different kingdoms. Similar forces drive microbial community establishment in both plant and animal hosts, including environmental factors, host genotype, nutrient availability, immune response, interactions with other microorganisms, etc. (15). Nonetheless, it has recently been shown that there are no abundant bacterial taxa shared between the microbiota in independently evolved, yet functionally related, organs in animals and plants (15). This suggests that the type of host determines the associated microbial community and that host factors play an important role in the successful colonization of eukaryotic hosts.

Although significant progress has been made in the last decades to identify bacterial genes and mechanisms required for host colonization (18–21), the extent to which the identified gene sets overlap or encode for similar pathways is not well investigated. Hence, our understanding on lifestyle-specific (detrimental versus non-detrimental) and/or host-specific (animal versus plant) colonization factors has remained fragmentary. Genome-wide analysis tools developed in recent years have greatly advanced our comprehension of the mechanisms determining the lifestyle of a bacterium and its ability to colonize a particular host. Acquiring this knowledge is the basis for the development of targeted manipulation strategies to deter pathogen infection or to promote colonization with mutualistic microbes, with the aim to improve human health and the health of plant resources.

## TRANSPOSON SEQUENCING: A POWERFUL TOOL TO STUDY GENE FUNCTION

Great advances in understanding biological processes result from determining the function of individual genes. One of the most common conventional approaches to identify the underlying genetic mechanisms involved in bacterial growth in a particular condition (including the colonization of a eukaryotic host) is to disrupt genomic regions and then screen the mutant library for a given phenotype (e.g., the loss of colonization ability) (22, 23). Transposons, which are short DNA fragments that are able to move within the genome (24), are one of the major tools that have been employed for mutagenesis in order to determine gene function and/or fitness (25). Tn*5* and *mariner* transposons have been used most frequently in Gram-negative bacteria because of their simple procedures and broad host range. In general, *mariner*-based transposons are favored because of their lack of insertion bias and because the construction of dense libraries that are saturated is more easily achieved (26). Tn*5* transposons enable greater insertion density and genomic resolution than *mariner* transposons. However, they have a preference for high GC regions, and there can be insertion bias that leads to wrong fitness assignment (27, 28).

In conventional mutagenesis approaches, the phenotype of each mutant is measured individually, which can require considerable amount of labor and time. Additionally, phenotype assessment is conducted in the absence of other strains, which can be misleading because the fitness of a mutant can be affected when it is grown in the context of the entire mutant population. Gene function discovery using traditional transposon mutagenesis and subsequent screening of individual mutants is very time-consuming and thus is often the rate-limiting step in linking genotype with phenotype. In the last years, sequencing technologies have progressed significantly, accelerating the screening process and the identification of genotype–phenotype links. One of the most powerful genome-wide tools to detect the genetic determinants involved in fitness in a certain condition is transposon insertion sequencing (TnSeq or TIS), consisting in the creation of a high-density transposon mutant library followed by challenging the library in a selective condition (29–32). Querying gene functions in bacteria by means of transposons has been revolutionized due to the evolution and price reduction of next-generation sequencing (NGS) techniques. Using NGS to detect the transposon insertion sites, the function affected in the targeted bacteria can be linked to the disrupted gene. The prevalence of each transposon junction is directly proportional to the fitness of the corresponding mutant in a given condition; such that low number of reads of a given gene corresponds with low number of strains carrying that mutation, which means that the mutant has a low fitness (31, 33, 34). Since NGS allows simultaneous sequencing of transposon insertion sites in large mutant collection, high-throughput genome-wide analysis is possible (35). The advent of these high-throughput sequencing technologies has greatly enhanced gene function and/or fitness discovery both *in vitro* and *in vivo*. They allow simultaneous assessment of the relative frequencies of pools of thousands of individual mutants after being challenged to specific selective conditions, and thereby the identification of loci that are important for bacterial fitness under that investigated environment (33). In contrast to conventional methods, TnSeq efficiently measures fitness in a population-dependent context in a less laborious and time-consuming manner. Compared to other genome-wide analysis tools, such as RNA-seq that explores transcriptional regulation of gene expression, TnSeq is an accurate predictor of gene mutation phenotypes. Since expression and fitness do not correlate well (33, 36), both TnSeq and RNA-seq are complementary approaches and should ideally be combined along with other methods, such as evolution experiments (to unravel the genetic changes that lead bacteria to bypass essentiality) (37), and metabolomics (to fully understand resource use in bacteria-host interactions) (38–40).

**Fig 2:**
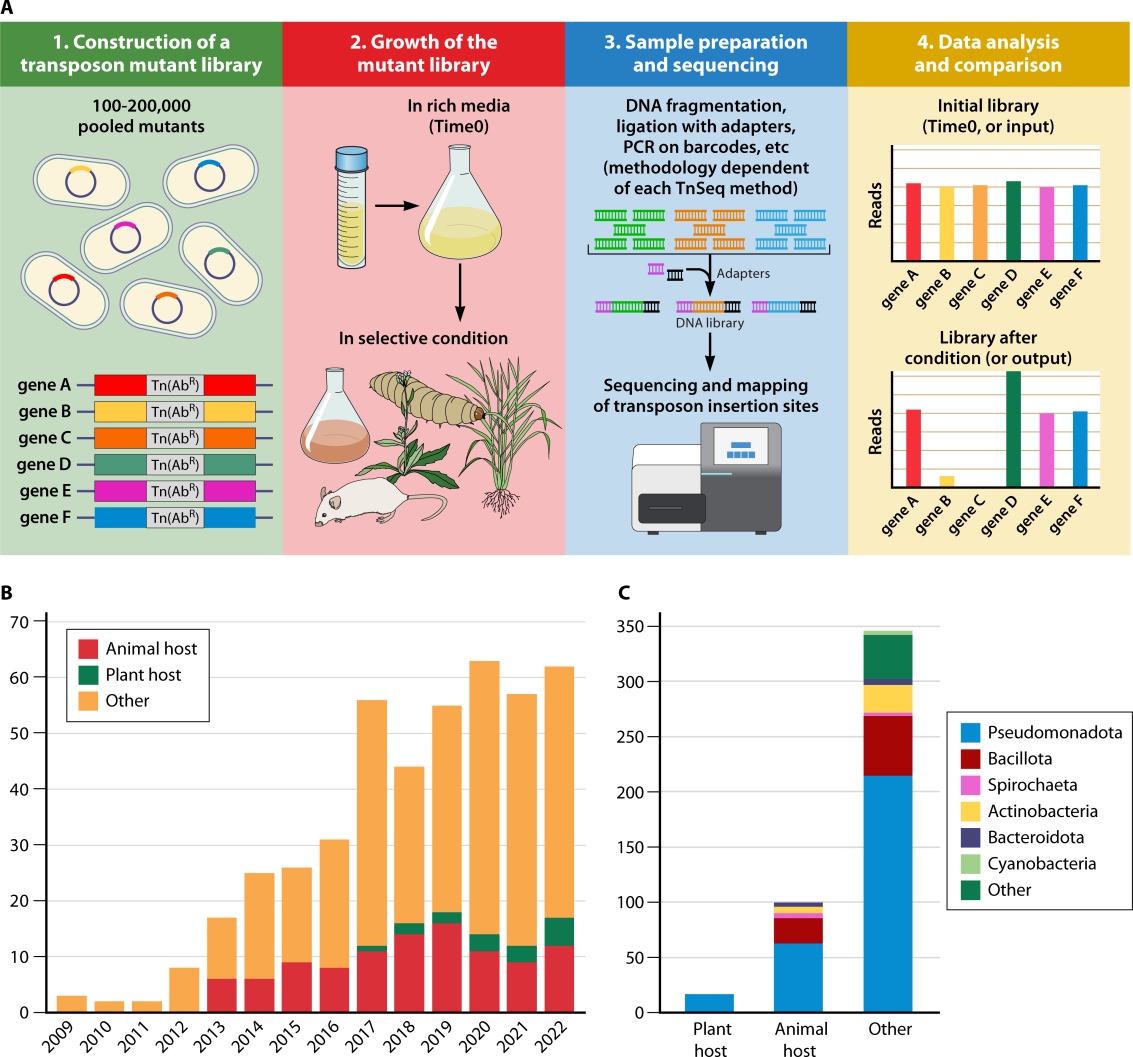
Use of TnSeq to assess gene function. (A) Outline of the TnSeq workflow. (B) TnSeq studies published per year since 2009. Data were downloaded from the Web of Science (date of search: 1 June 2023) and curated manually. In orange, *in vivo* animal host TnSeq studies. In green, *in vivo* plant host TnSeq studies. In yellow, other types of TnSeq studies (essential genome identification, *in vitro*, *ex vivo* models, protocols, analysis tools, reviews, etc.). (C) Number of different bacterial phyla targeted in *in vivo* plant host studies, *in vivo* animal host studies, and other type of TnSeq studies. The data used to construct these graphs were curated manually and can be found in Table S1.

A number of equivalent approaches and innovative variations of transposon insertion sequencing have been developed since the first reports in 2009, including TnSeq (31, 32), transposon-directed insertion site sequencing (TraDIS) (30), high-throughput insertion tracking by deep sequencing (HITS) (29), INSeq (41), transposon liquid enrichment sequencing-Seq (TnLE-seq) (42), randomly barcoded (RB) TnSeq (34), droplet TnSeq (dTnSeq) (43), restriction enzyme-mediated integration insertion sequencing (REMI-seq) (44), loxTnSeq (45), sorTnSeq (46), TRADISort (47), density-TRADISort (48), TnSeq-circle (49), and others. While most of them separate the mutants based on their ability of growing or not in a selective condition, other methods select them based on their physical properties, such as motility (50), density (related to capsule formation) (48), or fluorescence (related to altered efflux activity and uptake of a fluorescent DNA intercalating agent) (47). For simplicity, in this review we will refer to all the above approaches as TnSeq. While these methods differ in how the sequencing libraries are prepared and how to amplify the transposon–genome junction to identify the transposon insertion site, they are all conceptually similar and are largely shared (35, 51). Briefly, a saturated transposon mutant library typically contains enough mutants to cover the maximum number of genes in the genome of a given bacterium (i.e., around 50,000–200,000 mutants). Then, the library is grown in rich media. An aliquot of the library (called control, Time0, t0 population, input, etc.) is kept for genomic DNA (gDNA) extraction and sequencing. Next, the library is grown in a selective condition (e.g., *in vitro*, *ex vivo*, or *in vivo*), the mutants are harvested after growth for multiple generations, and their gDNA is extracted. The next step is to prepare the samples for sequencing. The methodology to do so differs according to each TnSeq approach used. Finally, the data are analyzed using different software, such as TRANSIT (52), Con-ARTIST (53), or TRADIS (54). These data analysis tools compare the relative abundance of mutants before (Time0, input, etc.) and after the growth in the selective condition (output) (Fig. 2A), and a fitness or fold-change value is calculated for each gene (33, 55). Genes that cannot tolerate mutations and are absolutely required for survival are often referred to as essential genes (non-viable transposon mutants). Underrepresented mutants after growth in the selective condition correspond to genes that provide a fitness benefit in the wild-type strain. They are usually called attenuated genes, depleted genes, important genes, or conditionally essential genes, and when mutated, they have a negative impact on fitness. Mutants that are enriched compared to the control are called overrepresented genes or detrimental genes for fitness (if mutated, they have a positive impact on fitness).

Since the first time that TnSeq was introduced in 2009 (29–32), a large number of *in vitro* and *in vivo* studies using this methodology have been conducted in various bacteria (Fig. 2B). TnSeq-related methods have even been successfully applied in other organisms, such as yeast (56), microalgae (57), archaea (58), and amoeba (44). For simplicity purposes, in this review we will focus only on the research conducted in bacterial species. The large majority of the TnSeq studies published so far (Table S1) deals with the identification of functions required for growth under *in vitro* conditions, while *in vivo* host colonization studies only constitute a small fraction (Fig. 2B). *In vivo* animal host studies are more abundant than *in vivo* plant host studies, for which only very few publications are available (Fig. 2B). Although more studies focus on bacteria with a pathogenic lifestyle, commensal and mutualistic bacteria have also been investigated (Table S1). Regarding the phyla targeted with these studies, most of them have been conducted in species of the phylum Pseudomonadota (Proteobacteria), other investigated bacteria belong to the phyla Bacillota (Firmicutes), Actinobacteriota (Actinobacteria), Bacteroidota (Bacteroidetes), Spirochaetota (Spirochaetes), and Cyanobacteria (Fig. 2C). This unbalance likely reflects the ease of genetic manipulation and the high cultivability of members of the phylum Pseudomonadota. Optimized transposon delivery vectors often do not exist for non-model organisms, and they are efficient only in easily culturable and genetically tractable species. A key challenge in the field in the upcoming years will be to develop tools that allow difficult-to-manipulate microbes of medical, industrial, and environmental importance to be assayed. Methods such as the “magic pool” approach (59) that optimize transposon delivery in non-model organisms have already started to address this issue.

Despite the many advantages and applications of TnSeq, as evidenced by the rapidly growing number of publications using it (Fig. 2B), the approach has also some limitations. It cannot report on genes coding for redundant functions and those coding for the synthesis of secreted components that could be shared as “public goods,” acting in the surrounding environment of the transposon mutant population beyond the cell containing the mutation (60). Examples of this are siderophores, quorum-sensing molecules, biosurfactants, hydrolytic enzymes, toxins, biofilm matrix material, etc. (61). Testing the entire population of mutants can mask cheaters present in the population that could have a different fitness if tested individually. A promising TnSeq methodology, dTnSeq, was recently developed to address this issue: microfluidic-facilitated encapsulation allows isolated growth of individual transposon mutants and consequently the identification of single-cell phenotypes without interference from other members of the mutant population (43). Thibault et al. (43) determined that 1%–3% of mutants in *Streptococcus pneumoniae* have a different fitness when grown in isolation versus in a pool of mutants. Another constraint of TnSeq is the impact that the conditions the library is constructed (media, temperature, etc.) may have. A gene might not have insertions if its mutants are at low abundance after recovery from the freezer, or if its mutants might grow poorly in the conditions that were used to generate the mutant library. Additional biases may be caused by polar effects of transposon insertions within an operon, although it has been shown that such biases are negligible in a number of microorganisms (31, 62, 63). Other limitations affecting the quantitative aspect of TnSeq are transposon insertion bias, the complexity of the libraries created, and technical sources of noise during DNA library preparation or during the amplification and sequencing of transposon junctions (27). Finally, although not a constraint *per se* but a major challenge when designing TnSeq experiments, is whether or not the selection condition will impose a “bottleneck” that will result in the stochastic loss of insertion strains regardless of their fitness (64). This is particularly important when *in vivo* models (33) are used for selection as discussed more thoroughly in "Experimental bottlenecks" below.

Notwithstanding these limitations, TnSeq is a powerful approach that has proven to be successful in hundreds of bacteria, supporting its utility as a functional genomic tool. Recent methodological advances have helped to mitigate the biases and constraints of TnSeq approaches (33, 34, 43). A future challenge will be to use TnSeq to answer increasingly complex and diverse biological questions (33). For this, we must move away from the homogeneously grown laboratory cultures to more sophisticated model systems that better mimic natural environments. A driving factor of this challenging expansion will be increasing the numbers of conditions and bacterial strains that can be simultaneously screened, building on RB-TnSeq (34) and similar approaches. This would be followed by the analysis and comparison of multiple data sets, for which it is necessary for the development of interactive visualization tools, data-sharing platforms, machine learning tools, and modelling to combine and extract knowledge from the ever-expanding number of TnSeq studies (65–67).

## HOST COLONIZATION STUDIES USING TNSEQ APPROACHES

### Practical considerations when conducting *in vivo* TnSeq studies

Despite considerable advances and development of extensions of TnSeq approaches since its introduction in 2009, challenges remain in applying the technique to *in vivo* infection models to identify host colonization genes. Experimental design choices can heavily influence the result of TnSeq assays. Apart from the general limitations of TnSeq discussed above, two main considerations should be made when interrogating colonization of living hosts by the aid of this methodology.

#### Experimental bottlenecks

In theory, only mutants with attenuated survival or growth abilities should decrease in frequency under selective growth conditions. However, insertion mutants can be lost during selective growth due to fitness-independent stochastic processes, such as population constrictions due to experimental bottlenecks. In these bottleneck events, the population size is drastically reduced, and the surviving cells will make up the new population but will have reduced genetic diversity. Random loss of transposon insertions can significantly increase variation in the data as well as lead to the wrongful identification of fitness genes.

The more complex an experiment is (e.g., *in vivo* versus *in vitro*), the higher the probability that bottlenecks will happen. Different trade-offs between realism and library complexity exist for different *in vivo* models (33), for which often bottlenecks have been reported (53, 64, 68, 69). For example, >99.9% of an infectious dose of *Vibrio cholerae* is lost during infection of a rabbit model host (70), and >99.9% of *Listeria monocytogenes* does not survive orogastric inoculation of mice (71). Many of these bottlenecks may result from the acidic pH of the stomach, which is a severe but non-specific infection barrier. Some authors claim that bottlenecks are not related to the *in vivo* model *per se* but mostly to the nature of the interaction between the bacteria and the host. For instance, stochastic population losses were observed for *Campylobacter jejuni* using a 2-week-old chicken colonization model, but not for *Salmonella enterica* serovar Typhimurium under the same experimental conditions (72, 73).

Stochastic population losses can be detected and quantified experimentally or considered computationally (27, 33, 74). Different experimental and computational approaches can be used to mitigate bottlenecks (27, 55). For example, bottleneck effects can be reduced experimentally by using sub-pools of transposon libraries (at the same dose) that are less complex, such that representatives of all mutants in the input have a higher chance of passing the bottleneck (32, 68). Another example is the modification of the experimental conditions with the aim to decrease the stochastic mutant losses. For instance, in *V. cholerae* infections, antacids can be supplied to the animal host (75) to minimize the stomach acid-mediated bottleneck, even though such treatment may also affect the fitness of specific mutants. Despite the value of these experimental procedures, bottleneck events in some *in vivo* models cannot be avoided and therefore need to be corrected during data analysis (27). For instance, computational approaches can be used to predict the likelihood that mutants in neutral genes disappear stochastically from a library and then remove them from the analysis to compensate for an accurate fitness interpretation (53).

#### Enrichment after harvesting the mutants from the host

For most conditions, collecting a large quantity of bacterial cells representing the entire mutant population is not an issue. However, in some *in vivo* experiments, the number of cells (thus their gDNA) recovered from the host can be a limiting factor for performing the TnSeq protocol. The recovered library can be enriched (outgrown, amplified) by cultivation. Some authors have done so in rich media after collecting the mutants from the host (40, 76, 77). This allows to increase the total mass of mutants, facilitating DNA recovery for subsequential steps. Although this procedure might introduce some artifacts resulting from differences in mutant *in vitro* growth rates, in principle, the relative abundance of each mutant should not be changed, as the mutants should not have a fitness deficit in rich media, in which libraries have been constructed.

We evaluated whether enrichment of mutants after *in vivo* assays had an effect on the determined fitness values of genes using two previously constructed RB-TnSeq mutant libraries of *Paraburkholderia phytofirmans* PsJN and *Paraburkholderia bryophila* 376MFSha3.1 (34, 78). For that, 10-day-old maize (*Zea mays*) plants were inoculated with each of the mutant libraries. Seven days post-inoculation, rhizosphere samples were harvested (79), and each individual sample was divided in two sub-samples: one was used for direct gDNA extraction, and the other one was subjected to a 16-h enrichment in rich liquid media (LB) previous to gDNA extraction. The results showed a tight correlation (*R* > 0.82) of the fitness values of all genes between the two procedures (i.e., direct gDNA extraction versus enrichment and then gDNA extraction), suggesting that a short outgrowth on rich media does not have a major impact on fitness determination in the bacteria tested. We hypothesize that it will likely be the same with other libraries since the mutants should not have a major difference in fitness in rich media (in which the library was constructed), and therefore their abundance should not be significantly altered. We suggest that this should be verified for each library, ideally before designing the experimental setup.

### TnSeq studies conducted *in vivo*

Independently of the type of host (animal versus plant) or type of interaction (detrimental versus non-detrimental), bacteria must deploy efficient colonization mechanisms to ensure survival in the host. It is worthy to clarify that what TnSeq really assess in *in vivo* models is entry and early colonization in the host as well as persistence or long-term colonization. In principle, it would be misleading to state that it measures just survival, since survival does not require growth, and most TnSeq methods require mutant growth to select for individuals with a fitness defect or advantage. Nevertheless, it is noteworthy to mention that some bacteria have the ability to survive in a growth-arrested state for long periods of time, and in these microorganisms, TnSeq could be adapted to select mutants using non-growth-based approaches (80). Along the same line, it can be misleading to state that it strictly measures virulence, since in many bacteria, virulence is related to the production of “public goods” (e.g., siderophores, quorum-sensing molecules, proteases, etc.) (61), and the corresponding synthesis genes cannot be detected by TnSeq (see limitations of TnSeq above). Although novel TnSeq methodologies have been developed that allow the identification of single-cell phenotypes like secreted virulence-related traits, such as dTnSeq (43), or the selection of mutant libraries under non-growth conditions (80, 81), they cannot easily be applied to *in vivo* models, and their utility in the identification of fitness traits in plant and animal models remains to be evaluated.

Since the development of the method (29–32), TnSeq has efficiently identified the mechanisms that are required for efficient host colonization in diverse types of hosts: plants (e.g., tomato, maize, rice, pea, bean, cauliflower, lettuce, and *Arabidopsis*) and animals (e.g., mice, rabbit, squid, bee, tick, moth, cattle, baboon, chicken, and turbot fish) (Table S1). TnSeq has also been conducted in bacteria with different lifestyles, like the human pathogen *Staphylococcus aureus* (82), the plant pathogen *Agrobacterium tumefaciens* (40), the opportunistic pathogen *S. pneumoniae* (83), the plant mutualistic species *Pseudomonas simiae* (84), or the insect symbiont *Snodgrassella alvi* (85), among others. Interestingly, some TnSeq studies have used different hosts in which a target bacterium develops disparate lifestyles (pathogenic and non-pathogenic) (40, 76). A major driver for the development of genome-wide approaches such as TnSeq has been the analysis of detrimental interactions of pathogens with their hosts. As a consequence, a large fraction of published studies investigated human pathogens, while considerably fewer studies investigated mutualistic interactions of non-detrimental bacteria with their hosts (Table S1). In all cases, the application of TnSeq unraveled previously known as well as novel colonization determinants. Another interesting point is that many of the host colonization studies conducted to date have been conducted on reductionist conditions, usually mono-culture infections in gnotobiotic hosts, even though many bacteria live in diverse multispecies communities. Recent evidence suggests that the gene set needed by a bacterium to survive in an environment drastically differs when it is alone or within a community (32, 82, 86). These results suggest that the composition and interactions in a complex bacterial community can change the selective conditions that prevail in an environment. The fact that the significance of some colonization genes depends on the presence of other bacteria should be taken into consideration when designing disease management tools that target fitness genes, since they could be ineffective in the presence of other microorganisms.

An emerging line of research is the comparative analysis of *in vivo* fitness factors in several hosts to identify a core set of colonization genes in contrast to host-specific genes (40, 87). Despite the rapidly increasing number of *in vivo* TnSeq studies that have been conducted, there is a lack of integration of the advances made in each study. For instance, a platform that allowed the comparison of TnSeq data on genes providing a fitness benefit for interactions of bacteria with their plant and animal hosts would be an important first step for the identification of specific factors required for colonization. Such type of comparative tool would provide insights into how to design novel strategies that aim at mitigating or improving colonization by both pathogenic and mutualistic microorganisms.

## INTERROGATING BACTERIA–HOST INTERACTIONS WITH THE AID OF TNSEQ

In an attempt to identify the bacterial genetic determinants providing a fitness benefit in different types of hosts (plant versus animal) and types of lifestyles/interactions (detrimental versus non-detrimental), we compared 58 data set of studies conducted in diverse bacteria using TnSeq approaches (Table 1). The proportion of studies conducted in each host type and interaction type is comparable. We used the lists of underrepresented or depleted genes that were presented by the author as being important for fitness. It is noteworthy to say that the cut-off criteria (log_2_-fold decrease and confidence level) were different but comparable among studies. Table S2 (https://figshare.com/articles/dataset/Supplementary_Table_S2_xlsx/25511503) contains the data sets used for analysis, showing the lists of genes that are important for colonization (i.e., genes that if mutated have a negative impact on fitness) in host colonization compared to a control, usually undefined rich media such as LB, although some authors used minimal media supplemented with different nutrients and vitamins. In case relevant data were missing in the original publications, such as the one-letter COG (88) code of the genes, the information was retrieved from databases, e.g., National Center for Biotechnology Information or eggnog (89), or provided by authors upon request.

**TABLE 1:** Selection of host colonization TnSeq studies

	Host and organ	Bacterial species	Bacterial phylum	Interaction^*a*^	Growth time in the host	% of genome important	Reference	Data in original publication
Plant host	Tomato plant root	*Agrobacterium tumefaciens* C58	Pseudomonadota	Non-detrimental	5 weeks	∼8.0%	40	Table S2
	Tomato plant stem	*Agrobacterium tumefaciens* C58	Pseudomonadota	Detrimental	5 weeks	∼4.1%	40	Table S2
	Maize root	*Agrobacterium tumefaciens* C58	Pseudomonadota	Non-detrimental	5 weeks	∼2.9%	40	Table S2
	Poplar stem	*Agrobacterium tumefaciens* C58	Pseudomonadota	Detrimental	5 weeks	∼4.0%	40	Table S2
	Duckweed	*Aquitalea magnusonii* H3	Pseudomonadota	Non-detrimental	7 days	∼1.8%	90	Suppl. Data 5
	Green foxtail root	*Azoarcus olearius* DQS4	Pseudomonadota	Non-detrimental	15 days	∼2.1%	91	Table S2
	Rice root	*Burkholderia vietnamiensis* LMG10929	Pseudomonadota	Non-detrimental	7 days	∼6.4%	92	Data Sets S3 and S4
	Potato plant root	*Dickeya solani* RNS08.23.3.1A	Pseudomonadota	Non-detrimental	42 days	∼2.0%	76	Table S2
	Potato plant stem	*Dickeya solani* RNS08.23.3.1A	Pseudomonadota	Detrimental	5 days	∼5.0%	76	Table S2
	Green foxtail root	*Herbaspirillum seropedicae* SmR1	Pseudomonadota	Non-detrimental	15 days	∼2.1%	91	Table S2
	Corn xylem	*Pantoea stewartii* subsp. *stewartii* DC283	Pseudomonadota	Detrimental	6 days	∼4.4%	93	Table S3
	Rice root	*Paraburkholderia kururiensis* M130	Pseudomonadota	Non-detrimental	7 days	∼3.8%	92	Data Sets S3 and S4
	Maize root	*Pseudomonas aeruginosa* PGPR2	Pseudomonadota	Non-detrimental	7 days	∼1.9%	94	Table S3
	*Arabidopsis* root	*Pseudomonas simiae* WCS417	Pseudomonadota	Non-detrimental	7 days	∼2.0%	84	Suppl. Data S1
	Common bean leaf	*Pseudomonas syringae* B728a	Pseudomonadota	Non-detrimental	2 days	∼0.6%	95	Table S5
	Common bean apoplast	*Pseudomonas syringae* B728a	Pseudomonadota	Non-detrimental	6 days	∼1.3%	95	Table S6
	Lima bean apoplast	*Pseudomonas syringae* B728a	Pseudomonadota	Non-detrimental	6 days	∼0.5%	95	Table S3
	Tomato plant stem	*Ralstonia pseudosolanacearum* GMI1000	Pseudomonadota	Detrimental	5 days	∼2.5%	96	Table S1
	Pea plant rhizosphere	*Rhizobium leguminosarum* bv. *viciae* Rlv3841	Pseudomonadota	Non-detrimental	5 days	∼2.3%	97	Data Set S1
	Pea plant root	*Rhizobium leguminosarum* bv. *viciae* Rlv3841	Pseudomonadota	Non-detrimental	5 days	∼2.8%	97	Data Set S1
	Pea plant root nodule	*Rhizobium leguminosarum* bv. *viciae* Rlv3841	Pseudomonadota	Non-detrimental	28 days	∼7.2%	97	Data Set S1
	Pea plant bacteroid	*Rhizobium leguminosarum* bv. *viciae* Rlv3841	Pseudomonadota	Non-detrimental	28 days	∼5.6%	97	Data Set S1
	*Medicago* root nodule	*Sinorhizobium meliloti* CL150	Pseudomonadota	Non-detrimental	42 days	∼15.7%	98	Data set EV3
	Cauliflower plant hydathode	*Xanthomonas campestris* pv. *campestris* 8004	Pseudomonadota	Detrimental	6 days	∼3.8%	99	Table S3
	Lettuce leaf	*Xanthomonas hortorum* pv. *vitians* LM16734	Pseudomonadota	Detrimental	10 days	∼3.2%	100	Table S2
Animal host	Mouse lung	*Acinetobacter baumannii* ATCC17978	Pseudomonadota	Detrimental	24 hours	∼4.5%	101	Table S1
	Greater wax moth	*Acinetobacter baumannii* AB5075	Pseudomonadota	Detrimental	4 hours	∼7.6%	102	Table S1
	Mouse abscess	*Aggregatibacter actinomycetemcomitans* 624	Pseudomonadota	Detrimental	3 days	∼11.2%	82	Table S1
	Mouse gut	*Bacteroides ovatus* ATCC8483	Bacteroidota	Non-detrimental	16 days	∼1.4%	103	Table S5
	Mouse gut	*Bacteroides thetaiotaomicron* ATCC29148	Bacteroidota	Non-detrimental	14 days	∼5.8%	104	Table S6
	Mouse lung and trachea	*Bordetella pertussis* UT25lux	Pseudomonadota	Detrimental	3 days	∼4.3%	105	Table S4
	Baboon lung	*Bordetella pertussis* D420	Pseudomonadota	Detrimental	7 days	∼4.7%	106	Table S3
	Tick larvae	*Borrelia burgdorferi* 5A18NP1	Spirochaetota	Non-detrimental	3–5 days	∼3.0%	107	Table 2
	Nematode	*Burkholderia cenocepacia* J2315	Pseudomonadota	Detrimental	24 hours	∼3.7%	108	Table S1B
	Mouse lung	*Burkholderia pseudomallei* JW280	Pseudomonadota	Detrimental	66 hours	∼8.7%	109	Table S1
	Mouse lung	*Brucella melitensis* 16M	Pseudomonadota	Detrimental	120 hours	∼3.5%	110	Table S2
	Piglet gut	*Campylobacter jejuni* M1cam	Pseudomonadota	Detrimental	5 days	∼7.4%	111	Table S1
	Chicken caeca	*Campylobacter jejuni* M1cam	Pseudomonadota	Detrimental	6 days	∼10.4%	112	Table S4
	Housefly	*Campylobacter jejuni* M1cam	Pseudomonadota	Non-detrimental	4 hours	∼2.9%	112	Table S4
	Turbot liver, spleen, and kidney	*Edwadsiella piscicida* EIB202	Pseudomonadota	Detrimental	14 days	∼4.6%	113	Table S1
	Rabbit gut	*Escherichia coli* EDL933	Pseudomonadota	Detrimental	2 days	∼3.9%	114	Table S6
	Mouse vagina	*Enterococcus faecalis* OG1RF	Bacillota	Non-detrimental	8 days	∼14.7%	115	Table S3
	Mouse spleen	*Francisella tularensis* subsp. *tularensis* SchuS4	Pseudomonadota	Detrimental	24 hours	∼8.9%	116	Table S4
	Greater wax moth	*Klebsiella pneumoniae* RH201207	Pseudomonadota	Detrimental	6 hours	∼2.8%	77	Table S5
	Cattle lung	*Mycobacterium bovis* AF2122/97	Actinobacteriota	Detrimental	6 weeks	∼3.7%	117	Table S3
	Mouse lung	*Mycobacterium tuberculosis* H37Rv	Actinobacteriota	Detrimental	8 days	∼1.1%	118	Fig. 1C
	Mouse gut	*Neisseria musculi* AP2365	Pseudomonadota	Non-detrimental	8 weeks	∼11.3%	119	Data S2
	Mouse oral cavity	*Neisseria musculi* AP2365	Pseudomonadota	Non-detrimental	8 weeks	∼12.2%	119	Data S2
	Mouse skin wound	*Pseudomonas aeruginosa* PAO1	Pseudomonadota	Detrimental	3 days	∼2.1%	120	Table S5
	Bee gut	*Snodgrasella alvii* wkB2	Pseudomonadota	Non-detrimental	5 days	∼17.4%	85	Data Set S1
	Mouse skin wound	*Staphylococcus aureus* HG003	Bacillota	Detrimental	4 days	∼11.2%	82	Table S1
	Mouse uterus	*Streptococcus agalactiae* CJB111	Bacillota	Non-detrimental	3 days	∼7.5%	121	Table S1
	Mouse cervix	*Streptococcus agalactiae* CJB111	Bacillota	Non-detrimental	3 days	∼5.6%	121	Table S1
	Mouse lung	*Streptococcus pneumoniae* TIGR4	Bacillota	Detrimental	24 hours	∼14.5%	122	Table S1
	Piglet brain	*Streptococcus suis* 10	Bacillota	Detrimental	5 hours	∼12.4%	123	Table S2
	Rabbit intestine	*Vibrio cholerae* C6706	Pseudomonadota	Detrimental	18 hours	∼10.0%	124	Table S4i and ii
	Squid hatchling	*Vibrio fischeri* ES114	Pseudomonadota	Non-detrimental	48 hours	∼9.5%	125	Table S1
	Rabbit intestine	*Vibrio parahaemolyticus* RIMD 2210633	Pseudomonadota	Detrimental	24 hours	∼4.8%	126	Table S3A

^*a*^ Type of interaction with the host was assigned according to whether disease symptoms in the given host were observed or not.

^*b*^ The lists of genes that provide a fitness benefit for host colonization in these studies can be found in Table S2 (https://figshare.com/articles/dataset/Supplementary_Table_S2_xlsx/25511503).

The studies in Table 1 were selected on the basis that they covered phylogenetically diverse bacteria and different host organisms and where the genomes of the strains were reasonably well annotated, thus disregarding works in which most of the identified genes were identified as belonging to COG category S (function unknown). TnSeq studies conducted in host-mimicking environments, such as *in vitro* or *ex vivo* in host tissues and fluids, like tomato xylem sap (127) or sputum (128), were not considered in this review. Another criterion for choosing these 58 data sets was that the percentage of genes important for host colonization should be similar. This should minimize the risk that the studies were biased due to bottlenecks, as the percentage of insertions that disappear from a library during selection has been suggested to serve as a proxy for bottlenecks in the experimental setup (55). Therefore, we excluded studies in which a high percentage (>20%) of genes was identified to be involved in host colonization, for instance (129), >60%. In the data sets of the chosen studies, on average, 5.7% of the total genome was found to provide a fitness benefit for host colonization (Table 1); this corresponded to 7.2% in the case of animal hosts and 3.8% in the case of plant hosts. Interestingly, this percentage was similarly independent of whether the interaction was detrimental or non-detrimental.

Comparison between TnSeq studies can be challenging due to the different procedures used by each author to calculate gene fitness and the format in which the data has been made available. Additionally, some authors use libraries grown in rich media (Time0, input, etc.) as a control, while others use minimal media or a host-mimicking fluid, which strongly influences the outcome. When using minimal media as a control, genes encoding anabolic pathways may disappear from the output list. However, these pathways are likely to be important for host colonization as they are needed to overcome nutrient limitations associated with the host niche. Regarding the use of rich media, it can also be very different in each study. For instance, the commonly used LB medium is a rich undefined medium composed of mixtures of yeast cell extracts and enzymatic digests of protein. The exact source and amount of each of the ingredients is often unknown and may vary according to the manufacturer. As stated above, the list of genes that provide a fitness benefit (i.e., genes with a negative fitness value in the TnSeq analysis) in the 58 selected studies (Table 1; Table S2 https://figshare.com/articles/dataset/Supplementary_Table_S2_xlsx/25511503) is all based on a comparison with nutrient-rich media, usually the same that was also used to construct the mutant library.

In an attempt to identify shared or unique pathways involved in animal versus plant host colonization and detrimental versus non-detrimental interactions, we classified genes according to functional categories, COG (88). Although COG is not the most informative gene classification method, it allows comparison among different species and may be sufficient to shed some light on the basic requirements for the colonization of different hosts by bacteria with different lifestyles.

**Fig 3:**
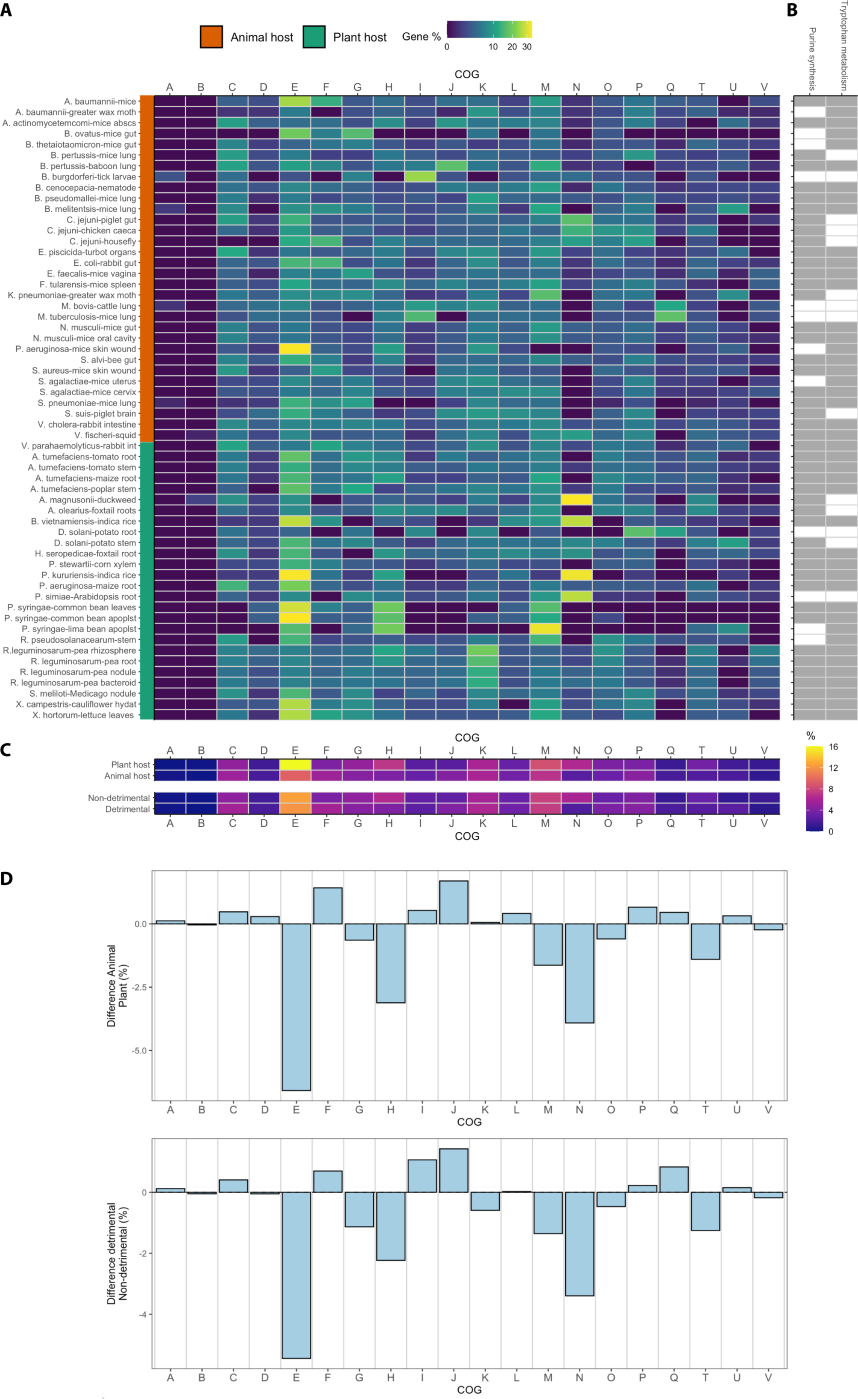
Overview of genes providing a fitness benefit for different interactions of bacteria with plant and animal hosts. (A) Percentage of genes for each COG categories identified as important for colonization of animal and plant hosts. For illustration purposes, square root of percentage values is presented. (B) Identification of genes required for purine biosynthesis (*purABCDEFHKLMN*) and tryptophan metabolism (*trpABCDEFGHRS* and *aroABCEGQ*) as important (grey) for host colonization in the different species. White color means no genes of the pathway were found to be important for colonization in the corresponding TnSeq study. (C) Average percentage of genes in each COG category involved in colonization, according to type of host and type of interaction. For illustration purposes, COG category S (corresponding to function unknown) is not shown. (D) Difference between average percentages of genes in each COG category involved in colonization, according to type of host and type of interaction. COG categories description: A = RNA processing and modification; B = chromatin structure and dynamics; C = energy production and conversion; D = cell cycle control, cell division, and chromosome partitioning; E = amino acid transport and metabolism; F = nucleotide transport and metabolism; G = carbohydrate transport and metabolism; H = coenzyme transport and metabolism; I = lipid transport and metabolism; J = translation, ribosomal structure, and biogenesis; K = transcription; L = replication, recombination, and repair; M = cell wall, membrane, and envelope biogenesis, N = cell motility, O = post-translational modification, protein turnover, and chaperonesl; P = inorganic ion transport and metabolism; Q = secondary metabolites biosynthesis, transport, and catabolism; S = function unknown; T = signal transduction mechanisms; U = intracellular trafficking, secretion, and vesicular transport; V = defense mechanisms.

When taking the bacterial host pairs independently (Fig. 3A) we could not detect a clear difference in the percentage of genes in each COG category that were important for animal versus plant colonization (Fig. 3A), which could point to a prevalence of a cell process for the colonization of a given type of host. We observed that in most of the studies considered in Table 1, pathways such as purine synthesis and tryptophan metabolism were important for efficient colonization (Fig. 3B), independently of the type of host or type of interaction. The results on the fitness benefit given by nucleotide biosynthesis for host colonization agree with previous reports. *De novo* purine synthesis has been described as the base of bacterial pathogenesis (130, 131) and of mutualistic interactions (132). Regarding tryptophan, it is one of the most expensive amino acids in terms of biosynthesis costs, which explains why among the 20 proteogenic amino acids, tryptophan is the least abundant residue in most proteins (133). Consequently, this aromatic amino acid is chemically precious, and its bioavailability is often limiting proliferation. The available data point toward low levels of tryptophan in both animal and plant hosts and to the need of synthesizing less abundant amino acids for efficient host colonization. Interestingly, depletion of intracellular tryptophan during invasion has been well documented as an anti-bacterial strategy in diverse animal hosts (134). These data suggest that this may as well be the case for plant hosts. The last common precursor to tryptophan is chorismate, produced via the shikimate pathway. Since the shikimate pathway is important for many organisms, it is an attractive target for antibiotics (135). One example of a chemical compound that is specifically targeting shikimic acid and disrupting the growth of bacteria, fungi, plants, etc. is the well-known herbicide glyphosate (136). Noteworthy, purine synthesis and tryptophan metabolism pathways may not appear as important for host colonization in some species if the genes involved are essential, meaning that mutations in those genes cannot be tolerated and therefore not being represented in the initial mutant library or if the initial mutant library was first selected on minimal media. This could lead to some TnSeq reports being contradictory to previous literature. It is the case for *Mycobacterium tuberculosis*, for which tryptophan synthesis had reportedly been identified as needed to colonize and produce disease in animals (137) but was not identified by TnSeq as involved in host colonization (117, 118). This could probably be due to the construction of the library in poor media (128, 129), in which tryptophan biosynthesis mutants would grow very poorly and thus may be lost during library preparation.

In an effort to facilitate the detection of differences and commonalities in COG categories involved in fitness benefit in animal versus plant host colonization and detrimental versus non-detrimental lifestyles, we calculated the average percentage of genes in each COG category for each type of host and type of interaction (Fig. 3C), as well as the difference between the average percentages of genes in each COG category involved in colonization according to type of host and type of interaction (Fig. 3D). When considering the type of host, the main differences between the animal versus plant studies were observed for the E (amino acid biosynthesis) and the N (cell motility) COG categories (Fig. 3C and D). On the basis of the data sets used for this analysis (Table 1), we conclude that genes involved in these two classes seem more important for plant colonization than for animal colonization (Fig. 3D, largest negative values). We reason that this difference in the amino acid class abundance might indicate a lower content and reduced availability of amino acids in plants compared to animal hosts and therefore a more stringent need of amino acid biosynthesis genes for efficient plant colonization (138). Simons et al. (139) proved that the amino acid concentration found in root exudate is insufficient to contribute substantially to the nutrition of rhizosphere bacteria. Interestingly, amino acids produced by plant-associated bacteria have been shown to regulate rhizosphere pH and as a consequence plant immunity (138). This highlights the relevance of amino acid synthesis by plant bacteria in order to modulate the host environment and thereby assure colonization. Regarding the difference in cell motility genes across the selected TnSeq studies, we hypothesize that flagellar motility is a key factor for the successful colonization of plant hosts but not for animal hosts. Animal colonizers such as *S. enterica* serovar Typhimurium, *Escherichia coli*, *L. monocytogenes*, and *Pseudomonas aeruginosa* do not require flagellar motility for host colonization (140). In contrast, in numerous plant–bacteria interaction studies the flagellum has been shown to play a crucial role for host colonization as well as for recognition by the host (141, 142). Consistent with the above, motility genes have been identified by TnSeq in plant but not animal colonization by *P. aeruginosa* (94, 120).

The main difference in the type of interaction (detrimental versus non-detrimental) was seen for the COG class N (cell motility) (Fig. 3C and D). This suggests that not many differences between detrimental and non-detrimental lifestyles have been identified by TnSeq. In fact, when calculating the absolute value of the sum of the differences observed (Fig. 3D), a value of only 17.7 is obtained for the detrimental versus non-detrimental interaction studies, while for the animal versus plant host studies, this value is of 24.6. This is probably due to the fact that the two types of interactions depend on common underlying mechanisms and most of the functions distinguishing both interactions are related to common goods, for which the TnSeq approach is blind. It has been argued that detrimental and non-detrimental interactions are similar in terms of persistence and colonization (3, 14). This suggests that despite the type of interaction of a given bacterium with its host, the nutritional requirements within a same host or host organ are comparable, and therefore, similar genes will be identified. The mere presence of a bacterium in a host usually does not kill the host until virulence factors are produced. However, as many virulence factors are shared goods, these can normally not be identified by TnSeq.

## CONCLUSIONS AND FUTURE DIRECTIONS

Bacterial species share numerous colonization factors, despite their different lifestyles (detrimental versus non-detrimental) and type of host (animal versus plant). Compared to other methods, in which gene function is studied one gene at a time, significant progress has been made thanks to genome-wide high-throughput technologies such as TnSeq. The rapidly increasing number of TnSeq experiments in diverse bacteria is proof of the interest raised by this technique in the scientific community. Despite the large number of TnSeq data sets available for host colonization, shared conclusions are often disregarded in the literature, and a global comparison with the aim to identify unique or shared traits for different types of hosts or types of interactions is lacking. The analysis presented in this review supports the idea that detrimental and non-detrimental interactions of bacteria with their hosts at least in part depend on common underlying mechanisms and that many of these functions are also independent of the type of host (animal versus plant). Differences in the availability of nutrients in each host niche appear to present the main selective forces for fitness gene identification. This is not only a consequence of the fact that the TnSeq methodology has limitations in the identification of virulence factors but also an underappreciation of the role of nutrient availability in the host during colonization (6, 143).

A future challenge will be to use TnSeq to answer increasingly complex and diverse biological questions. In order to bridge the gap between reductionist host colonization assays and more complex and natural environments, we must move toward more sophisticated model systems that better mimic what happens in natural hosts. Future TnSeq work to identify genes required for *in vivo* survival should consider that the selective forces in the host organism can also be influenced by host age, presence of chemical compounds, biotic and abiotic stresses, etc. (15, 32, 86, 103, 144). An important point to consider in the upcoming colonization studies is the fact that the host is often not colonized by a single strain but by a multispecies microbiome. For instance, it may be possible that a bacterium does not need certain nutrition genes when other members of the consortium provide them. This agrees with findings on how the gene set needed by a bacterium to survive in an environment drastically changes if alone or in the presence of other bacteria. Apart from the presence of other microbiota, we recommend that the dietary and nutritional statuses of the host are also taken into consideration in future research, since the availability of nutrients in a host could vary and would therefore strongly affect the identification of fitness genes. Finally, the creation of an online repository where TnSeq data could be deposited would facilitate data comparison and decision-making on which relevant pathways directly target in disease management strategies or when developing novel plant biostimulant strategies.

## SUPPLEMENTAL MATERIAL

Table S1 mbio.00390-24-s0001.xlsx Compilation of TnSeq studies since 2009.

ASM does not own the copyrights to Supplemental Material that may be linked to, or accessed through, an article. The authors have granted ASM a non-exclusive, world-wide license to publish the Supplemental Material files. Please contact the corresponding author directly for reuse.
